# Integrating Blinatumomab in the Frontline Treatment in B-Cell Acute Lymphoblastic Leukemia: A New Era in Therapeutic Management

**DOI:** 10.3390/jcm14062055

**Published:** 2025-03-18

**Authors:** Martina Canichella, Laura De Fazio, Matteo Molica

**Affiliations:** 1 Hematology, St. Eugenio Hospital, ASL Roma2, 00144 Rome, Italy; martina.canichella@aslroma2.it; 2 Department of Hematology-Oncology, Azienda Universitaria Ospedaliera Renato Dulbecco, 88100 Catanzaro, Italy; defalaura@gmail.com

**Keywords:** blinatumomab, frontline setting, B-cell acute lymphoblastic leukemia (B-ALL), Philadelphia chromosome-positive ALL (Ph+ ALL), measurable residual disease (MRD), allogeneic stem cell transplantation (allo-SCT), chimeric antigen receptor T cells (CAR-T)

## Abstract

Blinatumomab, a bispecific T-cell engager (BiTE), has shown substantial efficacy in treating both relapsed/refractory (R/R) Philadelphia chromosome (Ph)-positive and Ph-negative acute lymphoblastic leukemia (ALL). With its targeted mechanism of action, favorable safety profile, and ability to induce deep molecular remissions, blinatumomab is increasingly incorporated into frontline treatment regimens for B-ALL. Recently, the Food and Drug Administration (FDA) has approved its use in the frontline setting for Ph-negative ALL. In Ph-negative ALL, combining blinatumomab with intensive chemotherapy has resulted in superior measurable residual disease (MRD) clearance and improved long-term outcomes. In Ph-positive ALL, combination therapies involving tyrosine kinase inhibitors (TKIs), particularly ponatinib and blinatumomab, are challenging the traditional approach of allogeneic hematopoietic stem cell transplantation (allo-SCT). This review explores the current evidence supporting the frontline use of blinatumomab in newly diagnosed adults with B-ALL, its impact on treatment paradigms, and potential future directions, including novel combination therapies and the role of emerging immunotherapeutic approaches.

## 1. Introduction

Over the past three decades, the prognosis for patients with B-ALL has markedly improved. These advancements are primarily attributed to more refined risk stratification based on molecular characteristics, the adoption of MRD-guided treatment protocols, and the introduction of immunotherapy [1,2].

In particular, for Ph-negative B-ALL, improved outcomes have been correlated to the adoption of pediatric-inspired chemotherapy regimens. These regimens are characterized by dose-intensive chemotherapy, a shortened maintenance phase, and enhanced central nervous system (CNS) prophylaxis. Using these approaches, the MD Anderson Cancer Center (MDACC) study group reported a complete remission (CR) rate exceeding 90% and a 5-year overall survival (OS) rate of 40% [3,4]. Similarly, the GIMEMA (Gruppo Italiano Malattie Ematologiche dell’Adulto) trial LAL1913, which introduced a dose-dense intensive regimen, demonstrated superior outcomes compared with historical controls, with 3-year OS, event-free survival (EFS), and disease-free survival (DFS) rates of 66.7%, 57.7%, and 63.3%, respectively [5].

Since the early 2010s, monoclonal antibodies targeting CD19 and CD22 have been investigated in clinical trials for R/R B-ALL [6,7,8,9,10], leading to the approval of these agents as monotherapies in the salvage setting [9,10]. Among them, blinatumomab, a bispecific T-cell engager (BiTE) targeting CD19, was approved in 2014, while inotuzumab, an antibody-drug conjugate containing calicheamicin targeting CD22, received approval in 2017 [9,10]. As a result, therapeutic strategies have shifted toward combination regimens in both salvage and frontline treatment settings, contributing to improved survival outcomes.

In newly diagnosed Ph-negative B-ALL, Jabbour et al. conducted a phase 2 trial evaluating the combination of hyper-CVAD regimen (cyclophosphamide, vincristine, and dexamethasone) and blinatumomab. With a median follow-up of 37 months, the estimated 3-year relapse-free survival (RFS) was 73%, demonstrating promising efficacy [11]. Similarly, the GIMEMA LAL2317 trial showed favorable results, with 3-year OS and DFS rates of 71% and 66%, respectively, based on a median follow-up of 36.5 months [12]. The randomized phase III E1910 trial further assessed the benefit of incorporating blinatumomab into consolidation therapy for newly diagnosed Ph-negative ALL patients who achieved MRD negativity after induction. The addition of four cycles of blinatumomab significantly improved survival compared to chemotherapy alone, with a 3.5-year OS of 85% versus 68% in the control arm, corresponding to a 59% reduction in mortality risk [13].

In Ph-positive ALL, the introduction of TKIs in the early 2000s marked a revolution in treatment strategy, with imatinib being the first-in-class agent [14]. Initially, TKIs were combined with chemotherapy, resulting in significant toxicity and treatment-related mortality. To mitigate these risks, since 2004, all frontline GIMEMA protocols have adopted a chemotherapy-free induction approach based on TKIs and glucocorticoids (with CNS prophylaxis) [15,16,17,18]. The addition of blinatumomab has further improved treatment outcomes. The GIMEMA LAL2116 protocol introduced a frontline regimen combining blinatumomab with dasatinib, demonstrating high efficacy with minimal toxicity [19]. At a 53-month follow-up, the regimen showed durable responses, with DFS, overall survival OS, and event-free survival EFS rates of 75.8%, 80.7%, and 74.6%, respectively [20].

Building on this success, the next step involved combining blinatumomab with ponatinib, a potent pan-TKI that can overcome resistance associated with the T315I mutation. This combination was first evaluated in a phase II study conducted by MDACC, which reported high complete hematologic response (CHR) rates of 88%. The estimated 3-year PFS and OS rates were both 95% [21]. An update revealed that, with a median follow-up of 24 months, the molecular response by reverse transcriptase-polymerase chain reaction (RT-PCR) was 83%, and the rate of MRD negativity by next-generation clono-sequencing (NGS) was 98% [21]. Recently, preliminary results from the GIMEMA LAL2820 trial, which has recently closed enrollment, further demonstrated the potential of this combination [22]. This randomized phase III study compared ponatinib plus blinatumomab with imatinib plus chemotherapy in Ph-positive ALL. Notably, 110 patients (95%) achieved a CHR. At a median follow-up of 6.4 months, the estimated 18-month OS rate was 91.6%. Although the follow-up period was relatively short, these results are particularly encouraging and may suggest a potential shift in the future allocation of allo-SCT.

Looking ahead, key challenges include the incorporation of subcutaneous formulations of blinatumomab, which have shown promising results in the R/R B-ALL setting, and the evolving role of chimeric antigen receptor T-cell (CAR-T) therapy, which is currently approved for R/R Ph-negative B-ALL patients [23,24]. The recent approval of frontline blinatumomab for Philadelphia-negative B-ALL patients inspired this review, which summarizes the key clinical experiences with blinatumomab across different age groups.

## 2. The FDA Approval of Blinatumomab for First Line Setting

In June 2024, the FDA granted approval for blinatumomab to treat both adult and pediatric patients aged 1 month and older with CD19-positive, Ph-negative ALL during the consolidation phase, regardless of MRD status [25]. This decision was supported by data from the phase 3 ECOG-ACRIN E1910 study (NCT02003222) [13]. The primary endpoint of this trial was OS from randomization, comparing chemotherapy plus blinatumomab to chemotherapy alone in MRD-negative patients. A total of 224 patients aged 30 to 70 years with newly diagnosed Ph-negative ALL, who achieved MRD-negative remission following induction chemotherapy, were randomized equally into two groups. The control group received four cycles of consolidation chemotherapy alone. The experimental group underwent two cycles of blinatumomab followed by three cycles of chemotherapy, another cycle of blinatumomab, another chemotherapy cycle, and a final cycle of blinatumomab. During each blinatumomab cycle, patients received a continuous intravenous infusion for 4 weeks, followed by a 2-week break. Participants could also undergo allo-SCT at the discretion of the treating physician. Both groups then received standard prednisone, vincristine, 6-mercaptopurine, and methotrexate (POMP) maintenance chemotherapy for approximately 2 years to increase the likelihood of long-term disease remission [26].

After a median follow-up of 43 months, the study demonstrated a significant improvement in OS for patients randomized to the blinatumomab arm during consolidation compared to those who received chemotherapy alone. Median OS was not reached in either group. The 3-year OS and RFS rates were 85% and 80% for the blinatumomab group compared to 68% and 66% for the chemotherapy-only arm, respectively. While the trial was not designed to analyze subgroups, the greatest benefit was observed in patients aged 30 to 55 years.

Regarding toxicity, the blinatumomab arm experienced treatment-related non-hematologic toxicities at rates of 43% grade 3 (severe), 14% grade 4 (life-threatening), and 2% grade 5 (fatal). In the chemotherapy-only group, the rates were 36% grade 3, 15% grade 4, and 1% grade 5. As expected, neurological side effects were more common in the blinatumomab + chemotherapy group, affecting 23% of patients compared to 5% in the chemotherapy-only group, though most were manageable. In total, there were 17 deaths in the blinatumomab + chemotherapy arm, with eight due to relapse and nine from non-relapse causes, primarily infections. In the chemotherapy-only arm, there were 40 deaths: 31 from relapse, 7 from non-relapse causes (mostly infections), and 2 from unknown reasons.

The approval was also supported by the Study 20120215 (NCT02393859), a randomized, controlled, open-label, multicenter trial involving pediatric and young adult patients with Ph-negative ALL [27]. Patients were randomized 1:1 to receive either blinatumomab or the IntReALLHR2010 HC3 intensive combination chemotherapy as their third consolidation cycle. Randomization was stratified by age, MRD status at the end of induction, and bone marrow status following the second consolidation chemotherapy block. A total of 54 patients were assigned to the blinatumomab group and 57 to the chemotherapy group. The 5-year OS rates were 78.4% for the blinatumomab arm and 41.4% for the chemotherapy arm, resulting in an OS hazard ratio of 0.35 (95% CI: 0.17, 0.70). For RFS, the 5-year rates were 61.1% in the blinatumomab group compared to 27.6% in the chemotherapy group, yielding an RFS hazard ratio of 0.38 (95% CI: 0.22, 0.66).

## 3. Ph-Negative Adult ALL (Age up to 65 Years)

The prognosis of patients with Ph-negative ALL has progressively improved due to the adoption of pediatric-inspired chemotherapy regimens and enhanced risk stratification based on genetic features at diagnosis, along with MRD-driven treatment response. These advances have facilitated more precise indications for allo-SCT. In an effort to further improve outcomes without adding significant toxicity, blinatumomab has been explored in frontline therapy by several cooperative groups. The ECOG-ACRIN E1910 trial, which led to its approval in the first-line setting, was outlined above. Below, we summarize other key experiences, as presented in Table 1.

The phase II GIMEMA LAL2317 trial built upon the chemotherapy backbone of the previous GIMEMA LAL1913 study, adding two cycles of blinatumomab (administered during cycles 3 and 6) [12]. This trial enrolled 146 patients aged 18 to 65 years, with the primary endpoint being the MRD-negativity rate after the first blinatumomab cycle (time-point 3, TP3). After the induction phase, 131 patients (88%) achieved complete hematologic remission (CHR), and after the first blinatumomab cycle, the MRD-negativity rate was 93%. With a median follow-up of 36.5 months, the entire cohort exhibited an OS rate of 71% and DFS of 66%. OS differed significantly across age cohorts: 76%, 74%, and 49% for patients aged 18–40, 40–55, and >55 years, respectively (*p* = 0.00013). The overall cumulative incidence of relapse (CIR) was 27.5%, with higher rates observed in the Ph-like subgroup, a well-recognized poor prognostic group (see below). This study demonstrated improved outcomes for Ph-negative ALL patients compared to historical controls, even for those older than 55 years. However, the Ph-like subgroup continued to experience poor outcomes despite blinatumomab treatment. Toxicity data from this study have not yet been released.

The phase 2 GRAALL-2014 study investigated the therapeutic effect of blinatumomab in high-risk B-ALL subsets, including those with a Ph-like profile, KMT2A rearrangements, IKZF1 deletion, and persistent MRD positivity following the induction phase [28]. Patients who were candidates for allo-SCT received blinatumomab for a minimum of 4 weeks prior to transplant, while all other patients underwent five cycles during the consolidation and maintenance phases. A total of 95 patients, with a median age of 35 years (range 18–60 years), who were in CHR after induction and the first consolidation, were enrolled to receive blinatumomab from week 12. Final risk classification identified 45 patients as high-risk (HR) and 49 as very-high-risk (VHR). Pre-blinatumomab MRD was <0.01% in 56% of evaluable patients (49/88). Forty patients (42%) proceeded to allo-SCT. The median number of blinatumomab cycles for those not proceeding to allo-SCT was four cycles (range 1–5). After blinatumomab, complete MRD response was achieved in 61/82 (74%) patients. MRD response to blinatumomab was lower in patients with high pre-blinatumomab MRD levels but was not impacted by age, white blood cell count (WBC), or genomic subgroups. With a median follow-up of 20 months, the 18-month DFS and OS rates were 78.8% and 92.1%, respectively. Patients with VHR disease had significantly worse DFS (68.8%) compared to those with HR disease (90.6%; *p* = 0.018). This DFS difference was abrogated by censoring patients at transplant (VHR 88.1% vs. HR 90.6%, *p* = 0.10). Other factors significantly associated with better DFS included the DUX4/ERGdel subgroup, low pre-blinatumomab MRD, and complete MRD response after blinatumomab [28].

A single-arm, phase 2 trial conducted at MDACC investigated a first-line combination of hyper-CVAD followed by blinatumomab and, later, after 38 patients were enrolled, the addition of a lower dose of inotuzumab (InO) to reduce chemotherapy toxicity [29,30]. The initial cohort of 38 patients (median age 37 years) was treated with hyper-CVAD plus sequential blinatumomab (four courses, with an additional three courses during the maintenance phase with the POMP schema). The CHR rate was 100%, and the MRD-negativity rate was 97%, with a 3-year OS rate of 81%. Following the amendment, 25 patients (median age 24 years) received the combination of InO. The CHR rate was 100%, and the MRD-negativity rate was 91%, with an estimated 1-year OS of 100%. In the entire cohort, the CHR rate was 100%, irrespective of the Ph-like features, and the MRD-negativity rate was 95%, with no early deaths observed. Although the follow-up was short, these results suggest that a frontline immunochemotherapy regimen (blinatumomab + chemotherapy) can overcome the negative impact of certain genetic alterations.

The early introduction of blinatumomab has been implemented with the intent to decrease chemotherapy intensity and burden. A phase 2 study by the Australasian Leukemia and Lymphoma Group (ALLG) evaluated reduced-intensity chemotherapy in combination with blinatumomab [31]. Patients received low-intensity chemotherapy with cyclophosphamide, vincristine, and dexamethasone for debulking, followed by 7 days of blinatumomab. Subsequently, patients received three alternating cycles of blinatumomab and part B cycles of hyper-CVAD, followed by 2 years of maintenance therapy for subjects not proceeding to allo-SCT. All patients attained CHR, and of 26 evaluable patients, 70% had achieved an MRD response after cycle 1B, and 83% had achieved an MRD response by the end of cycle 2B. The estimated 24-month EFS and OS were 62% and 69%, respectively. Overall, the combination of blinatumomab with chemotherapy was well tolerated and efficacious, with high rates of CHR and MRD response.

## 4. Ph-like Subgroup

The term “Philadelphia-like” or “BCR/ABL1-like” was first introduced by Den Boer et al. in 2009 to describe a B-ALL subtype with a poor prognosis and a genomic profile resembling Ph-positive ALL but without the t(9;22) chromosomal translocation [32]. This entity was officially recognized in the 2022 World Health Organization (WHO) classification and by the International Consensus Classification [33,34].

Two major molecular signatures have been associated with the Ph-like profile: CRLF2 rearrangement, which is the most common, and non-CRLF2 rearrangements [35,36]. The latter includes various lesions such as ABL-class rearrangements, JAK2, and/or EPOR rearrangements as well as mutations of FLT3, NTRK3, PTK2B, BLNK, and RAS. To identify Ph-like cases, different techniques can be used. The gold standard is gene-expression profiling (GEP), but it is not suitable for routine diagnostic use. Therefore, other methods such as flow cytometry, PCR, and FISH have been developed to detect Ph-like cases [37].

Ph-like ALL is found in approximately 25% of adolescents and young adults (AYA; ages 15–39 years) and in about 20% of adults [35,36,37,38]. This subgroup exhibits resistance to standard intensive chemotherapy, persistent high levels of MRD positivity in CHR, and poor OS rates of 20–30% [39,40,41]. Due to their poor response to conventional chemotherapy, alternative therapeutic approaches have been explored for Ph-like cases, including the integration of TKIs and immunotherapies such as monoclonal antibodies, T-cell engagers, antibody-drug conjugates, and CAR-T-cell therapy. These approaches have shown promising potential in improving remission rates. Furthermore, ongoing clinical trials are investigating personalized treatment algorithms to refine risk-adapted strategies and enhance long-term survival [42]. Below, we summarize the most relevant studies regarding the use of blinatumomab in this setting.

Jabbour et al. conducted a post hoc analysis of the TOWER study to evaluate the impact of the Ph-like signature on the efficacy of blinatumomab in adults with R/R ALL [43]. The TOWER was a phase III trial comparing blinatumomab (*n* = 271) with standard-of-care chemotherapy (SOC; *n* = 134) in a cohort of R/R B-ALL patients. The blinatumomab arm showed a better median OS compared to the SOC group (7.7 months vs. 4.0 months, *p* = 0.01). A total of 142 cases were analyzed by RNA sequencing at MDACC to detect genomic alterations suggestive of a Ph-like signature. Of these, 101/142 (71%) were treated with blinatumomab, and 41/142 (29%) were treated with SOC. RNA sequencing identified 15 patients (11%) with Ph-like ALL—9 in the blinatumomab arm and 6 in the SOC arm. The median OS for Ph-like patients treated with blinatumomab was comparable to that of non-Ph-like patients, but it was significantly longer than that of Ph-like patients treated with SOC. The safety profile was consistent across both Ph-like and non-Ph-like patients, with cytokine release syndrome (CRS) and neurotoxicity being the most common adverse events, though their incidence did not differ significantly between the groups. Thus, this post hoc analysis suggests that blinatumomab may overcome the poor prognosis associated with Ph-like status.

The previously mentioned GIMEMA LAL2317 trial (see above) enrolled 31 patients identified as Ph-like by a BCR/ABL1-like predictor [12,39]. Of the 30 patients who relapsed, 10 were Ph-like positive. Among patients who achieved MRD negativity at time-point 3 (TP3), the CIR was 42.5% for Ph-like cases compared to 17.5% for the remaining patients. These data suggest that the combination of blinatumomab and chemotherapy did not fully overcome the negative impact of the Ph-like signature, which may require the addition of TKIs for more effective treatment.

Overall, the treatment of Ph-like ALL remains an unmet medical need, as outcomes are heterogeneous. A combination of chemotherapy, targeted therapy with TKIs, and immunotherapy are under investigation, and ongoing studies will determine whether they represent an optimal approach for managing these patients. At present, frontline allo-SCT remains strongly recommended for Ph-like cases and is considered the only potentially curative option.

## 5. Older Patients with Ph-Negative ALL (>65–70 Years)

The incorporation of immunotherapy—specifically blinatumomab and/or InO—into the frontline treatment strategy for older adults with B-ALL has garnered significant attention due to its promising efficacy and favorable safety profile. Older patients with B-ALL generally experience poorer outcomes with current chemotherapy-based treatments, with 5-year OS rates ranging from 10% to 20%. This is largely due to the higher incidence of adverse genetic factors and reduced tolerance to intensive chemotherapy regimens [44,45,46]. Even with age-adjusted and dose-reduced treatment protocols, early mortality rates remain significantly high. In Table 2, we summarize the most important studies conducted in this setting and provide further discussion below.

The MDACC conducted a study evaluating the combination of (dose-reduced hyperfractionated cyclophosphamide, vincristine, and dexamethasone) and InO for older patients with newly diagnosed Ph-negative B-cell ALL. The maintenance phase was initially planned to include monthly POMP courses for up to 3 years, but this was later modified by study amendment to include twelve POMP courses and four blinatumomab cycles (one blinatumomab cycle after every three POMP cycles) [46,47,48]. To improve outcomes, blinatumomab was added as consolidation therapy. The study enrolled 64 patients aged 60 and older, who received four cycles of mini-hyper-CVD with InO, followed by four cycles of blinatumomab, and maintenance therapy consisting of twelve cycles in total. Results showed that 98% of patients achieved CHR, and the MRD-negativity rate reached 77% after the first cycle and 94% at any point during therapy. The 60-day mortality rate was 3%, with no early deaths. The 3-year continuous remission and OS rates were 76% and 54%, respectively. Stratified by age, patients aged 60–69 years had a 3-year continuous CHR rate of 69% and an OS rate of 63%, while those aged 70 and older had rates of 87% and 42%. The lower survival in patients aged 70+ was mainly due to higher death rates while in CHR.

The SWOG1318 phase II trial evaluated blinatumomab followed by POMP maintenance in older patients (≥65 years) with Ph-negative B-ALL [49]. Patients received one or two cycles of blinatumomab in induction until CHR or CR was achieved. Afterward, they received three cycles of blinatumomab post-remission therapy, followed by 18 months of POMP maintenance. MRD was evaluated by flow cytometry. The study enrolled 29 patients with a median age of 75 years (range 66–84). The overall response rate was 66%, with 92% of responders achieving MRD negativity. The 3-year OS and DFS estimates were both 37%. At 3 years, 34% of patients were alive, marking a significant improvement over historical data. The 3-year DFS was 37%. No baseline factors were linked to treatment response.

The ALLIANCE A041703 study, a phase II trial, evaluated the use of InO induction followed by blinatumomab consolidation in older adults with newly diagnosed Ph-negative B-ALL [50]. Patients who achieved a reduction in marrow blasts by ≥50% or cellularity ≤20% after Induction IA proceeded to Induction IB, which consisted of InO at 0.5 mg/m^2^ on days 1, 8, and 15 of a 28-day cycle, followed by Induction IC with a higher dose of InO (0.8 mg/m^2^). Those who did not achieve cytoreduction during Induction IA proceeded directly to Course II, which consisted of blinatumomab. The primary endpoint was 1-year EFS. Thirty-three eligible patients were treated with a median age of 71 years (range 60–84). After a median follow-up of 22 months, the 1-year EFS was 75% (95% CI, 61–92%). Twelve patients experienced events, including nine relapses, two deaths in remission, and one death without achieving remission due to respiratory failure associated with sinusoidal obstruction syndrome of the liver. The 1-year OS was 84% (95% CI, 72–98%), with nine patients having died, six of whom died following relapse.

The GMALL group studied the use of sequential blinatumomab and chemotherapy in patients aged 56–76 years with B-ALL [51]. Patients who achieved CHR or partial remission (PR) with initial chemotherapy received blinatumomab, while those with induction failure underwent a second induction cycle followed by blinatumomab. Consolidation therapy consisted of three additional cycles of blinatumomab, followed by standard maintenance therapy for up to 2 years. Among 33 patients evaluable after the initial induction phase, 85% responded, 9% experienced treatment failure, and 29% achieved a molecular response. One-third of patients with failure after the first induction achieved CHR after the second induction, resulting in an overall CHR rate of 83%. Additionally, 82% of patients who achieved CHR experienced a molecular response following blinatumomab treatment. The OS at 1 year was 100% for patients aged 55–65 years and 66% for those over 65. The 1-year DFS for all patients was 89%.

The phase 3 randomized controlled Golden Gate Study enrolled patients aged ≥55 years (or 40–<55 years with severe, pre-defined comorbidities) with newly diagnosed Ph-negative B-ALL [52]. The study randomized patients to receive either blinatumomab alternating with low-intensity chemotherapy or standard chemotherapy. The investigational arm included two cycles of blinatumomab during induction, two cycles during consolidation, and three cycles during maintenance, alternating with chemotherapy. In the safety run-in phase, 10 patients were treated in the investigational arm. The treatment was well tolerated, with no early deaths reported. The regimen also demonstrated efficacy, with all enrolled patients achieving CR and 90% having an MRD response of <10⁻⁴ after the first induction cycle. Overall, these findings suggest that early administration of blinatumomab in elderly patients with B-ALL is well tolerated and effective, given the high MRD response rate, minimal treatment-related mortality, and promising short-term survival results.

## 6. Ph Negative ALL-Pediatrics

The 5-year OS rate for pediatric B-ALL has now exceeded 90%, but this rate has remained stable for nearly two decades. These findings suggest that with conventional treatment strategies, chemotherapy intensity has reached its tolerability threshold. Consequently, further improvements in survival outcomes and the reduction in treatment-related toxicities will require the development and integration of innovative therapeutic approaches. Immunotherapy, in particular, has garnered significant interest in the treatment of pediatric B-ALL [53]. Here, we summarize key clinical studies that have explored the use of blinatumomab in pediatric B-ALL patients.

The randomized phase 3 clinical trial previously described aimed for EFS in children with high-risk first-relapse B-ALL following a third consolidation course with either blinatumomab or consolidation chemotherapy, prior to undergoing allo-SCT [27]. The trial enrolled patients between November 2015 and July 2019, with data cutoff on 17 July 2019. A total of 108 patients (median age: 5.0 years [IQR, 4.0–10.5]) in CHR were randomized to receive either one cycle of blinatumomab (15 μg/m^2^/day via continuous intravenous infusion over 4 weeks) or chemotherapy as their third consolidation therapy. Enrollment was terminated early due to the observed benefit of blinatumomab, following a pre-specified stopping rule.

After a median follow-up of 22.4 months (range 8.1–34.2), the incidence of events was lower in the blinatumomab group compared to the chemotherapy group (31% vs. 57%; log-rank *p*  < 0.001). Deaths occurred in 8 patients (14.8%) in the blinatumomab group compared to 16 patients (29.6%) in the chemotherapy arm. The minimal residual disease (MRD) remission rate was higher in the blinatumomab group (90% [44/49] vs. 54% [26/48]). Additionally, blinatumomab demonstrated superior safety, with fewer serious adverse events (24.1% vs. 43.1%) and grade ≥3 adverse events (57.4% vs. 82.4%) compared to chemotherapy.

In the open-label, single-arm expanded access study (RIALTO), 110 children (aged >28 days to <18 years) with R/R B-ALL were enrolled and received up to five cycles of blinatumomab administered via continuous infusion [54]. The incidence of grade 3 or 4 cytokine release syndrome (CRS) was low (1.8%), as was the occurrence of neurotoxicity (3.6%). No fatal adverse events related to blinatumomab were reported. The median OS was 14.6 months (95% CI: 11.0–not estimable), with a significant difference between MRD responders (not estimable) and MRD non-responders (9.3 months; hazard ratio: 0.18; 95% CI: 0.08–0.39). Among patients achieving CHR after two cycles, 73.5% proceeded to allo-SCT. The 1-year OS probability was significantly higher for patients who underwent allo-SCT (87% vs. 29%).

The use of blinatumomab as part of frontline treatment in pediatric B-ALL was evaluated in a phase 3 clinical trial conducted by the Children’s Oncology Group [55]. This study aimed to assess the use of blinatumomab as a consolidation phase for intermediate- and high-risk patients with first relapse. A total of 208 patients (median age 9 years) were randomized, with 118 (57%) completing the assigned therapy. After a median follow-up of 2.9 years, the 2-year DFS was 54.4% in the blinatumomab group compared to 39.0% in the chemotherapy group. The 2-year OS was 71.3% in the blinatumomab group versus 58.4% in the chemotherapy group. Serious adverse events in the blinatumomab group included infection (15%), febrile neutropenia (5%), sepsis (2%), and mucositis (1%) compared to higher rates of infection (65%), febrile neutropenia (58%), sepsis (27%), and mucositis (28%) in the chemotherapy group.

Li et al. conducted a retrospective analysis of clinical data from pediatric B-ALL patients who received blinatumomab treatment at the Hematology & Blood Diseases Hospital of the Chinese Academy of Medical Sciences between August 2021 and October 2023 [56]. A total of 35 patients (mean age: 9.9 ± 4.2 years) were included in the study, divided into three groups: R/R, minimal residual disease clearance (MC), and chemotherapy intolerance (IC). Adverse events included grade 1–2 CRS in 57.1% of cycles, with 84.4% of these being grade 1 CRS. Grade 4 immune effector cell-associated neurotoxicity syndrome (ICANS) occurred in 1.8% of cycles. The median follow-up time was 5.7 months for the R/R group, with 1-year OS and EFS rates of 40.0% ± 21.9% and 33.3% ± 19.2%, respectively. Both the MC and IC groups had 100% EFS. These findings are consistent with prior studies, suggesting that blinatumomab is safe and effective for selected patients with R/R B-ALL, MRD clearance, and chemotherapy intolerance.

Finally, the phase 3 randomized trial AALL1731 presented at the 2024 American Society of Hematology (ASH) Congress evaluated blinatumomab as part of frontline treatment in pediatric B-ALL [57,58]. The study enrolled 1440 children, with 718 randomized to receive chemotherapy plus blinatumomab and 722 to receive chemotherapy alone. After a median follow-up of 2.5 years, the 3-year DFS and OS were 96.0% ± 1.2% and 98.4% ± 0.9%, respectively, for patients receiving blinatumomab, compared to 87.9% ± 2.1% and 97.1% ± 1.1% in the chemotherapy-only group. Treatment-related toxicity, including cytokine release syndrome, seizures, and sepsis, was low in the blinatumomab group. However, non-fatal sepsis and central venous catheter infections were more common in the blinatumomab arm among intermediate-risk patients (14.8% vs. 5.1%; *p* < 0.001). These findings support the use of blinatumomab in the frontline setting, with potential to become the new standard of care for pediatric B-ALL. In Table 3, we present a summary of the results from these representative studies.

## 7. Ph-Positive ALL

Ph-positive ALL is the most common subtype of B-cell ALL in adults, accounting for 20–25% of all B-ALL cases. The incidence of Ph-positive ALL increases with age, and although it is rare in children, it affects more than 50% of patients older than 50 years [59]. In Ph-positive ALL, two key therapeutic advancements have significantly improved patient prognosis: the introduction of TKIs and the subsequent incorporation of blinatumomab [15]. The combination of chemotherapy and BCR::ABL1 TKIs, such as imatinib (2000), dasatinib (2006), and ponatinib (2010), has led to substantial improvements, with CHR rates exceeding 90% and 5-year OS rates approaching 50%. However, this approach has been associated with considerable toxicity, particularly in older patients [15].

To address the challenges of Ph-positive ALL, the GIMEMA group adopted a chemo-free strategy, starting in 2004, based on TKIs, glucocorticoids, and CNS prophylaxis. The results showed a CHR rate of 90–100% and a 5-year OS rate of 40–45%, with reduced toxicity and no induction-related mortality [15,16,17,18]. With the advent of immunotherapy, blinatumomab demonstrated efficacy and promising results in the R/R Ph-positive ALL setting, and it began to attract attention for its potential in frontline treatment.

In the setting of Ph-positive ALL, Rambaldi et al. conducted a propensity score analysis comparing outcomes of 45 relapsed cases treated with blinatumomab to 55 patients receiving standard of care (SOC) [60]. The blinatumomab group showed superior CHR rates (36% vs. 25%) and OS rates compared to SOC. Moreover, the final analysis of the ALCANTARA study by Martinelli et al. confirmed the sustained efficacy of blinatumomab in R/R Ph-positive ALL patients [61]. Among 45 patients, 16 (36%) achieved CHR within two cycles. After a median follow-up of 16.1 months, the median RFS was 6.8 months, and the median OS was 9.0 months. Notably, MRD negativity was achieved in 14 of the 16 patients who attained CHR. These findings established blinatumomab as a highly effective option for R/R Ph-positive ALL, supporting its use in frontline settings.

The first chemo-free frontline trial combining blinatumomab and dasatinib was conducted by the GIMEMA group under the LAL2116 D-ALBA protocol [19]. Among 63 enrolled patients, the CHR rate was 98%. At the end of induction with dasatinib alone (day 85), 29% of patients achieved a molecular response, increasing to 60% after two cycles of blinatumomab. These results translated into impressive clinical outcomes: with a median follow-up of 18 months, the OS and disease-free survival (DFS) rates were 95% and 88%, respectively. Molecular analyses indicated worse outcomes for patients with IKZF1plus aberrations (deletions involving *IKZF1* with *CDKN2A/CDKN2B* or *PAX5*). Long-term follow-up at 53 months confirmed DFS, OS, and EFS rates of 75.8%, 80.7%, and 74.6%, respectively [20].

Building on these successes, the next step was the introduction of ponatinib in combination with blinatumomab. Ponatinib, a pan-BCR::ABL1 inhibitor, emerged as the most effective in inducing deep molecular responses, including MRD negativity, and overcoming resistance due to the T315I mutation. Short et al. conducted a phase II trial at MDACC evaluating five cycles of blinatumomab combined with ponatinib in 44 patients [62]. The complete molecular remission (CMR) rates were 64% after one course and 85% after treatment completion. Using NGS with a sensitivity of 10⁻⁶, MRD negativity was confirmed in 88% of cases [60]. Subsequently, the GIMEMA study group designed a phase III trial (LAL2820) comparing blinatumomab and ponatinib to an imatinib-plus-chemotherapy arm [22]. Preliminary results from the experimental arm were presented at the 66th ASH meeting. With a median follow-up of 6.4 months (range 0.1–32.3), the CHR rate was 98%, and the estimated 18-month OS was 91.6%. Table 4 illustrates the results of studies using blinatumomab in the frontline setting for Ph-positive ALL patients.

Paradoxically, these advancements have complicated the role of allo-SCT in Ph-positive ALL. The decision to proceed with allo-SCT is now reserved for specific cases, guided by biological characteristics at diagnosis (e.g., IKZF1plus signature) and early MRD response evaluation [63].

## 8. Incorporating Immunotherapy and CAR-T into the Treatment Strategies of B-ALL

As discussed above, the incorporation of blinatumomab into first-line treatment has resulted in significantly improved outcomes across different age groups and B-cell subsets. The current NCCN Clinical Practice Guidelines in Oncology (NCCN Guidelines^®^) recommend blinatumomab as part of frontline therapy across all groups of adult patients with newly diagnosed Ph-negative B-ALL [64]. Similarly, the emerging promising therapeutic approach in Ph+ ALL is the chemo-free one based on TKI plus blinatumomab, although this combination is not available outside of clinical trials. In the future, we reasonably expect a combined use of targeted therapies in order to reduce the chemotherapy. In this setting, the combination of blinatumomab and InO showed promising results, as we previously mentioned, in the ALLIANCE A041703 [50]. Indeed, Jabbour et al. reported the results of combination of InO with mini-hyper-CVD with or without blinatumomab in B-ALL newly diagnosed patients [65]. This phase 2 study enrolled 80 patients aged 60 years or older. With a median follow-up of 92.8 months, the arm that received blinatumomab showed better PFS and OS, resulting in a 5-year PFS of 41.8% and 5-year OS of 40.9%. In an older population, the promising results derived from reduction chemotherapy and the addition of InO and blinatumomab together with the favorable safety profile was translated into improved overall outcome. This strategy will also be applied in the setting of younger patients with the aim to induce deeper response. Finally, the role of the emerging cellular therapy with CAR-T and its role in immunotherapy sequencing is in development. Recently, the results of Felix, a phase 1b-2 multicenter study, tested the safety and efficacy of obe-cel in adults (≥18 years of age) with R/R B-ALL. The main cohort was represented by patients with morphological disease, while another cohort contained MRD-positive cases. Overall, 153 patients were enrolled, and CAR-T cells were successfully delivered in 146 patients (95.4%); 127 patients (83.0%) received at least one obe-cel infusion, and of these, 120/127 (94.5%) received both doses. The median age was 47 years (20–81), and patients had received a median of two prior lines of therapy (2–6), while 52% were refractory to the last treatment. Indeed, 41.7% of patients had previously received blinatumomab, 31.5% InO, and 16.5% both. Overall, remission occurred in 77%, with CR in 55% and CRh in 21%. Additionally, 62/68 patients were evaluable for MRD, and 58/62 (94%) achieved MRD-negativity status after obe-cel infusion. For about 127 patients who received at least one obe-cel infusion (median follow-up, 21.5 months), the median EFS was 11.9 months, while the median OS was 15.6 months. With regard to safety profile, grade 3 CRS occurred in 2.4% of the patients, while ICANS developed in 7.1% of cases. This study has highlighted the potential role of CAR-T cells in patients with R/R B-ALL, particularly in those with low burden and no extramedullary involvement. Consequently, in November 2024, the FDA approved obe-cel for the treatment of adult patients with R/R B-ALL. To fully understand the therapeutic impact of CAR-T cells in the management of both Ph-negative and Ph-positive B-ALL, further clinical trials evaluating their use in frontline settings are required. Moreover, the outcomes will need to be directly compared with those achieved with blinatumomab.

## 9. Discussion

The incorporation of blinatumomab into frontline treatment protocols has truly revolutionized the therapeutic approach to B-ALL, significantly improving outcomes across various patient subsets. Its efficacy in both Ph-positive and Ph-negative B-ALL, particularly in achieving MRD negativity, has solidified its position as a pivotal component of modern treatment regimens. MRD clearance continues to be one of the most powerful predictors of long-term survival in B-ALL, and blinatumomab has shown a remarkable ability to overcome the negative prognostic impact of persistent MRD positivity, offering patients a much-needed advantage in their treatment journey [9,11,12,15,27,46,48].

In Ph-negative adult B-ALL, the integration of blinatumomab into frontline regimens has significantly improved MRD clearance rates, translating into better long-term outcomes. These findings underscore the potential of blinatumomab to modify the natural course of the disease and suggest that its early use may represent a new standard of care for adults with Ph-negative B-ALL [13]. For elderly patients, where the toxicity of intensive chemotherapy often limits treatment options, blinatumomab has emerged as a well-tolerated alternative. Studies combining blinatumomab with reduced-intensity chemotherapy—and, in some cases, with InO—have demonstrated encouraging results in this population. This approach allows for sustained disease control while minimizing the risk of severe adverse events [47]. It is particularly relevant in Ph-negative ALL, where treatment decisions must carefully balance efficacy with tolerability to optimize both survival and quality of life.

For Ph-positive ALL, the treatment paradigm has undergone a major shift with the introduction of TKIs and their combination with blinatumomab. Historically, this subgroup was associated with a poor prognosis, with allo-SCT representing the only potential option for long-term survival in eligible patients. However, emerging data now suggest that combining third-generation TKIs, such as ponatinib, with blinatumomab can achieve deep molecular remission, potentially altering the role of allo-SCT in selected patients [22,62]. Moreover, the identification of specific genetic lesions, such as IKZF1plus, and monitoring MRD at key time points may further refine patient selection for transplantation, paving the way for a more personalized treatment approach. Despite these advancements, several challenges persist in optimizing the prognosis of B-ALL patients. In the Ph-like subgroup, the efficacy of blinatumomab remains controversial, highlighting the need for novel combinatorial strategies. The integration of TKIs or other targeted agents alongside blinatumomab and chemotherapy could improve outcomes in this high-risk population, but further studies are necessary to determine the optimal treatment sequence and combinations.

Finally, in pediatric B-ALL, the latest phase 3 trial demonstrated that incorporating blinatumomab into frontline treatment significantly improved outcomes, particularly in intermediate- and high-risk patients [27]. These findings support the integration of blinatumomab as a new standard of care in this population.

Overall, these changes raise important questions regarding the role of allo-SCT in the era of immunotherapy. While transplantation has traditionally been considered essential for high-risk B-ALL, the improved survival outcomes achieved with blinatumomab and TKIs have prompted a reassessment of its necessity, particularly for patients achieving deep molecular responses. The decision to proceed with allo-SCT must now be carefully considered, balancing potential risks with the molecular risk factors and the patient’s response to therapy. In addition, the development of a subcutaneous formulation of blinatumomab marks a significant advancement in the field [66]. Preliminary data from early-phase clinical trials in R/R adult Ph-negative ALL suggest that this formulation preserves efficacy while improving patient tolerability. If these findings are validated in larger studies, subcutaneous blinatumomab could expand treatment access, particularly in outpatient settings, and help address some of the logistical challenges associated with continuous intravenous infusion [67]. Moreover, its potential impact on high-risk subgroups, such as Ph-like ALL, remains an area of ongoing investigation.

In conclusion, while blinatumomab has already revolutionized the management of B-ALL, continued research is crucial to optimize its use. Future studies should aim to refine treatment algorithms, identify biomarkers predictive of response, and explore novel combinatorial approaches to enhance long-term outcomes. As the landscape of B-ALL treatment continues to evolve, these efforts will help tailor therapies to individual patient needs, improving survival rates and minimizing treatment-related toxicities.

## 10. Conclusions and Future Directions

The integration of blinatumomab into first-line therapy has undeniably enhanced response rates and survival outcomes while significantly reducing relapse rates in both Ph-positive and Ph-negative ALL across all age groups. This therapeutic advancement has brought substantial improvements, and with the anticipated introduction of the subcutaneous formulation, patient convenience and treatment efficacy could be further enhanced. This shift holds great promise in offering a more accessible and potentially more effective option for pediatric and adult patients alike.

Moreover, an exciting and rapidly advancing area of research is the use of CAR-T cell therapy in B-ALL. This innovative approach is showing considerable potential, especially in managing MRD-positive cases. CAR-T therapy may serve as a bridge to allo-SCT for carefully selected patients, expanding the therapeutic toolkit and refining outcomes for patients with high-risk or R/R disease [67]. The increasing success of these combined strategies could redefine treatment paradigms and offer hope for further improving long-term survival and quality of life for B-ALL patients.

## Figures and Tables

**Table 1:** Most representative studies with blinatumomab in first-line in Ph-negative ALL adult patients up to 60–65 year.

Study	Author Year	Study Design	N. of Patients	Age (yrs) Range	Grade ≥ 3 CRS	Grade ≥ 3 Neurotoxicity	CR Rate	MRD-Negative Patients	Induction Dead	OS	RFS/DFS
ECOG-ACRIN E1910	Litzow et al. 2024 [13]	CHT vs. CHT plus blinatumomab based on MRD status	224	51 (30–70)	14%	N/A	81%	N/A	N/A	85% (3 yrs)	RFS 80% (3 yrs)
GIMEMA LAL 2317	Chiaretti et al. 2023 [5]	Sequential blinatumomab following early and late consolidation	146	41 (18–65)	N/A	N/A	90.4%	73% (after early 96% (following first consolidation) blinatumomab)	N/A	83.8% (12 mo)	DFS 71.6% (12 mo)
MDACC	Jabbour et al. 2022 [11]	CHT followed by blinatumomab consolidation	38	37 (17–59)	3%	11%	100%	76% (induction) 95% (any time over therapy course)	N/A	85% (3 yrs)	RFS 84% (3 yrs)
GRAALL-2014	Boissel et al. 2021 [28]	Blinatumomab for consolidation and maintenance	95	35 (18–60)	0%	6%	N/A	74%	N/A	92.1% (18 mo)	DFS 68.8% (18 mo)

Ph: Philadelphia; ALL: acute lymphoblastic leukemia; CHT: chemotherapy; MRD: measurable residual disease; yrs: years; CRS: cytokine release syndrome; CR: complete remission; RFS: relapse-free survival; DFS: disease-free survival; N/A: not available; mo: months.

**Table 2:** Most representative studies with blinatumomab in first-line in Ph-like patients.

Study	Author Year	Study Design	N. of Patients	Age (yrs) Range	Grade ≥ 3 CRS	Grade ≥ 3 Neurotoxicity	CR Rate	MRD-Negative Patients	Induction Dead	OS	EFS/DFS
	Ashouri et al. 2024 [40]	Blinatumomab consolidation before allo-HSCT	268	43 (18–80)	N/A	N/A	91.0%	47.4%	N/A	88.4% (2 yrs)	EFS 5.8% (2 yrs)
	Wu et al. 2024 [41]	Combination of blinatumomab and a TKI in the frontline setting	19	27 (16–60)	N/A	N/A	90%	57%	N/A	100% (13 mo)	DFS 91.7% (13 mo)
TOWER	Jabbour et al. 2021 [43]	Post hoc analysis of blinatumomab versus CHT in R/R patients	142	40.8 ± 17.1	N/A	N/A	33%	50%	N/A	40% (12 mo)	N/A

Ph: Philadelphia; CHT: chemotherapy; R/R: relapse/refractory; MRD: measurable residual disease; OS: overall survival; yrs: years; CRS: cytokine release syndrome; CR: complete remission; EFS: event-free survival; DFS: disease-free survival; N/A: not available; mo: months.

**Table 3:** Most representative studies with blinatumomab in first-line in Ph-negative ALL-elderly (>65–70 years).

Study	Author Year	Study Design	N. of Patients	Age (yrs) Range	Grade ≥ 3 CRS	Grade ≥ 3 Neurotoxicity	CR Rate	MRD-Negative Patients	Induction Dead	OS	DFS/EFS
SWOG1318	Advani et al. 2022 [49]	Single-agent blinatumomab induction and consolidation followed by CHT maintenance	29	75	3%	3%	66%	N/A	N/A	37% (3 yrs)	DFS 37% (3 yrs)
ALLIANCE A041703	Wieduwilt et al. 2023 [50]	Sequential InO induction and blinatumomab consolidation	33	71 60–84)	N/A	N/A	85% (induction IA/B/C) 97% (course II)	N/A	N/A	84% (1 yr)	EFS 75% (1 yr)
GOLDEN GATE	Jabbour et al. 2022 [52]	Blinatumomab alternating with low-intensity induction CHT	10	69 (57–77)	0%	0%	100%	90% after induction cycle; 100% after induction cycle 1 2	N/A	76% (3 yrs)	N/A
GMALL BOLD TRIAL	Goekbuget et al. 2021 [51]	Blinatumomab in sequence with induction CHT	33	65 (56–76)	N/A	N/A	76% (after induction)	N/A	6%	100% (1 yr)	DFS 89% (1 yr)
	Kantarjian et al. 2018 [47]	Hyper mini-CVAD with InO, followed by consolidation and maintenance with blinatumomab	52	68 (60–81)	N/A	N/A	98%	94%	N/A	54%	EFS 59% (29 mo)

Ph: Philadelphia; ALL: acute lymphoblastic leukemia; CHT: chemotherapy; MRD: measurable residual disease; OS: overall survival; yrs: years; CRS: cytokine release syndrome; CR: complete remission; EFS: event-free survival; DFS: disease-free survival; InO: inotuzumab; N/A: not available; mo: months.

**Table 4:** Most representative studies with blinatumomab in first-line in Ph-negative ALL pediatric patients.

Study	Author Year	Study Design	N. of Patients	Age (yrs) Range	Grade ≥ 3 CRS	Grade ≥ 3 Neurotoxicity	CR Rate	MRD-Negative Patients	Induction Dead	OS	EFS/RFS
	Locatelli et al. 2021 [27]	CHT vs. blinatumomab as consolidation before allo-HSCT	108	5 (28 days–18 years)	N/A	N/A		90%	N/A	80% (24 mo)	EFS 66.2% (24 mo)
RIALTO	Locatelli et al. 2022 [54]	Blinatumomab in R/R B-ALL prior to allo-HSCT	110	8.5 years (0.4–17)	1.80%	3.60%	63%	57%	N/A	60% (14.6 mo)	RFS 50% (11.5 mo)
AALL1331	Brown et al. 2021 [55]	Blinatumomab versus CHT in post reinduction	208	9 (6–16)	1%	1%	N/A	75% (Blinatumomab group)	N/A	71.3% (2 yrs)	RFS 54.4% (2 yrs)
	Li et al. 2024 [56]	Retrospective analysis of blinatumomab use in R/R, MRD-positive or CHT-intolerant patients	35	9.9 (0.6–16.4)	N/A	1.8%	N/A	80%	N/A	40.0% ± 21.9% (1 yr)	EFS 100% (7.1 mo)
AALL1731	Gupta et al. 2024 [58]	Phase 3 randomized trial CHT alone or CHT plus blinatumomab	1440	median 4.3 years	N/A	N/A	N/A	N/A	N/A	3-year OS 98.4% in blinatumomab arm vs. 97.1% in CHT	3-year DFS 96% in blinatumomab arm vs. 87.9% in CHT

Ph: Philadelphia; ALL: acute lymphoblastic leukemia; CHT: chemotherapy; MRD: measurable residual disease; OS: overall survival; R/R: relapse/refractory; yrs: years; CRS: cytokine release syndrome; CR: complete remission; EFS: event-free survival; RFS: relapse-free survival; allogeneic-stem cell transplantation; N/A: not available; mo: months.

## Data Availability

Not applied for this review.
